# The Risk Factors and Neonatal outcomes of Isolated Single Umbilical Artery in Singleton Pregnancy: A Meta-analysis

**DOI:** 10.1038/s41598-017-07053-7

**Published:** 2017-08-07

**Authors:** Xiaohua Luo, Shanshan Zhai, Na Shi, Mei Li, Shihong Cui, Yajuan Xu, Limin Ran, Lidan Ren, Teng Hong, Rui Liu

**Affiliations:** 1 Department of Obstetrics and Gynaecology, The Third Affiliated Hospital of Zhengzhou University, Zhengzhou, 450052 Henan China; 2 Department of Obstetrics and Gynaecology, the People’s Hospital of Zhengzhou, Zhengzhou, 450052 Henan China

## Abstract

The current meta-analysis aims to evaluate the risk factors and neonatal outcomes of isolated Single Umbilical Artery (iSUA) in singleton pregnancy. Standard Mean Difference (SMD) or Weighted Mean Difference (WMD) was pooled for the maternal age, gravidity and parity, neonate birth weight and Apgar score one and five minutes after birth. We also pooled the odds ratios (ORs) at 95% confidence intervals (CIs) for maternal smoking status, the rate of neonate delivery before 37 or 34 weeks, Cesarean section (CS), the rate of being admitted to neonatal intensive care unit (NICU) and the serious adverse neonate outcome. Results show that maternal primigravidity [OR: −0.082, CI (−0.152, −0.011), p = 0.023] and female sex of the neonate [OR: 0.805, CI (0.673, 0.963), p = 0.017] were associated with higher risks of iSUA. As compared to normal neonates, the neonates with iSUA had lower birth weight, worse Apgar score, increased risk of delivery before the normal gestational age, increased rate of CS due to fetal distress, increased rate of admission to NICU and prolonged NICU stay. However, no difference in neonatal mortality was observed. Maternal primigravidity and female neonate might associate with increased risk of iSUA. Identification of iSUA is of great importance for prenatal diagnosis and may improve neonatal outcomes.

## Introduction

The normal umbilical cord contains two arteries and one vein (three-vessel cord). Single Umbilical Artery (SUA) is characterized by the absence of either the left or right umbilical artery^1^. This malformation has a reported incidence of up to 1% of pregnancies. The SUA incidence is 3–4 times higher in multiple pregnancies than in singleton pregnancy^2^. The most widely accepted causes for this anomaly are primary agenesis and/or later thrombotic atrophy^3^.

Previous studies have suggested many risk factors for iSUA, such as maternal age^4, 5^, maternal smoking status^1, 6^, ethnicity^4^, multiparity and multiple gestation^1, 7^. However, the results were inconsistent, possibly due to the limited sample size, varying geographic regions or differences among research subjects. For example, a study from Tulek *et al*. suggested that neither maternal age nor preterm delivery were associated with the development of iSUA^8^. Most cases of SUA were not associated with the presence of other malformations, in which chromosomal alterations were usually not present. However, certain obstetric complications were often observed in iSUA cases, such as fetal growth restriction and increased perinatal mortality^5, 9, 10^. Clinical relevance of iSUA was evaluated after birth in a prospective cohort study by Chetty-John *et al*.^4^. No significant difference in physical growth or neurological development was observed^4^. There was also no evidence of increased need for admission to neonatal intensive care units^11^. Leung and Robson^12^ noted that neonatal low birth weight was observed in around 28% of all SUA cases. Burshtein *et al*.^10^ reported that iSUA in fetus was a risk factor of Apgar score, whereas no relation was observed between the fetal iSUA and Apgar score in other studies^4, 8^. Neonates with SUA and iSUA had increased rates of prematurity, growth restriction and adverse neonatal outcomes^1^. Studies also indicated that risks of some adverse neonatal outcomes, such as low birth weight, preterm delivery and low Apgar score, were increased in cases with SUA^13, 14^.

In this study, we performed a meta-analysis to clarify the association of iSUA with the neonatal outcomes and the associated risk factors.

## Results

### Study description

**Figure 1:**
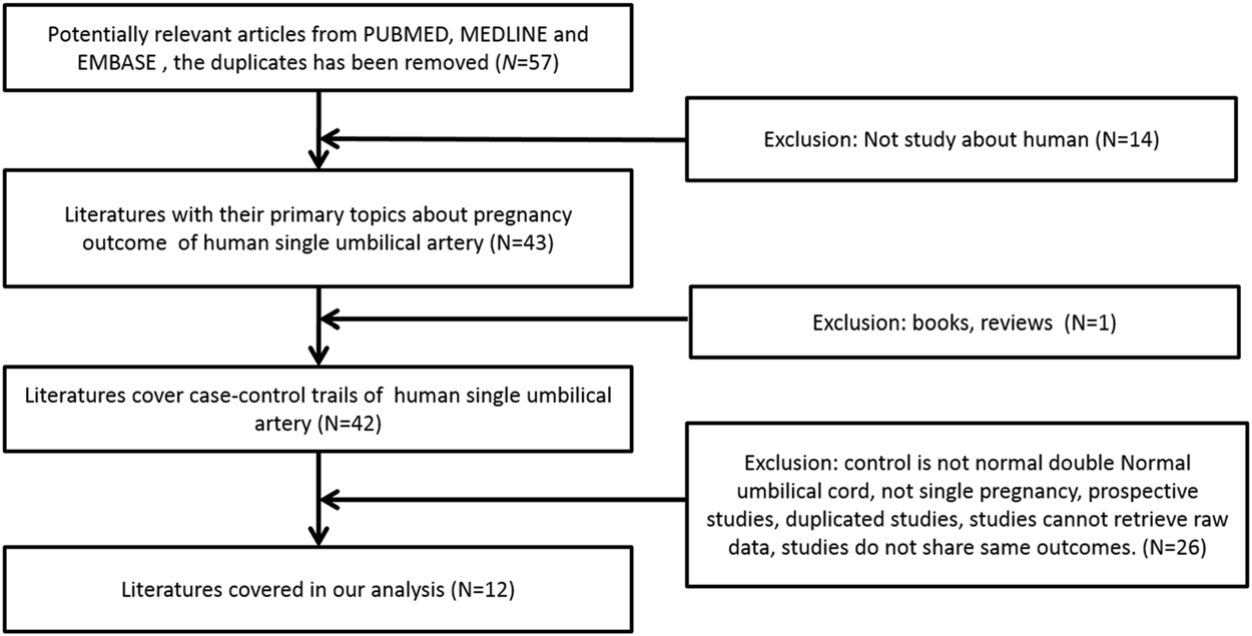
Flow chart of study selection and description of exclusion criteria for the mete-analysis.

A total of 57 relevant studies were retrieved from PUBMED, EMBASE and MEDLINE. We excluded the books, reviews and studies which are unrelated to humans, studies in which the control groups were not normal umbilical cord fetuses, studies in which case groups were not isolated SUA and studies of which the raw data could not be retrieved. For overlapped studies, only the ones with the most extensive results were included. After carefully examining all the literatures, a total of 12 qualified studies was included for the final meta-analysis. The data collection flow chart is shown in Fig. 1. The characteristics of 12 qualified studies are summarized in Table 1.

**Table 1:** Summary of studies and the risk factors of isolated SUA included in the meta-analysis.

Study	Population	Type of article	Number (case/control)^*^	Maternal age	Maternal gravidity	Maternal parity	Maternal smoking status
Predanic(2005)	American	retrospective, case-control study	84/84	Y	—	—	NR
Bombrys(2008)	American	retrospective, case-control study	255/289	Y	Y	N	NR
Mu(2008)	Taipei	retrospective, case-control study	14/28	N	N	N	NR
Horton(2010)	American	retrospective cohort study	68/68	Y	NR	NR	NR
Hua(2010)	American	retrospective cohort study	392/63655	N	Y	N	Y
Burshtein(2011)	Russia	retrospective cohort study	243/194566	N	—	—	NR
Mohamed (2013)	Saudi Arabia	retrospective cohort study	159/35026	N	N	N	NR
Ashwal(2014)	Israel	retrospective cohort study	91/182	N	—	—	N
Caldas(2014)	Brizil	retrospective, case-control study	134/759	N	NR	NR	NR
Doğan(2014)	Turkey	retrospective cohort study	77/95	N	N	N	NR
Tülek(2015)	Turkey	retrospective cohort study	93/100	N	N	N	Y
Mohamed(2015)	Austria	retrospective cohort study	136/500	NR	NR	NR	N

*Case/control = isolated SUA*/*normal umbilical artery. NR: no refer. “‑”: not available; Y: indicates that the factor is a risk of isolated SUA in this study. N: indicates the factor is not a risk of isolated SUA in this study.

### The risk factors of iSUA

**Figure 2:**
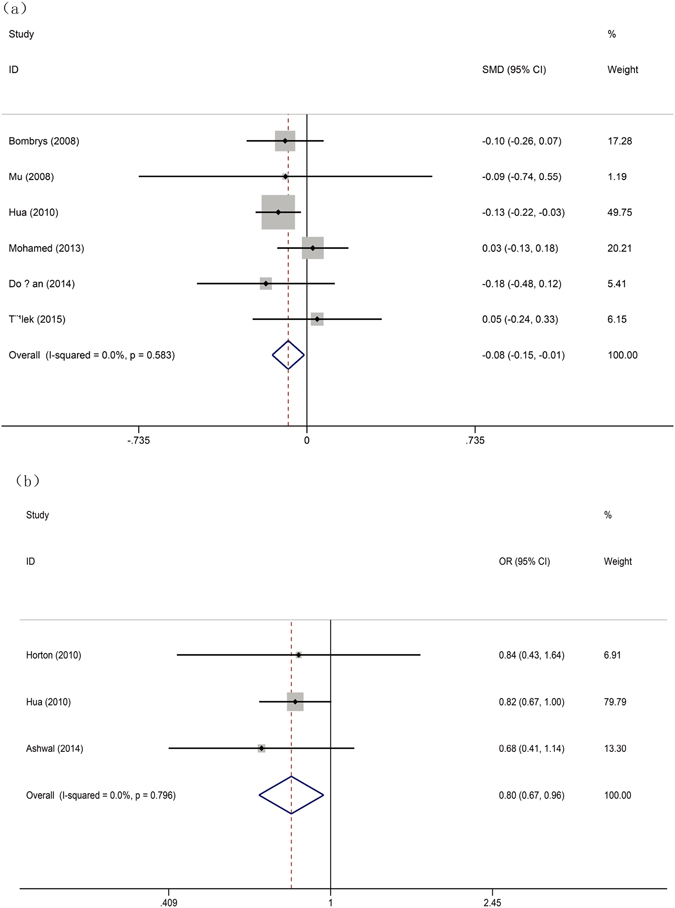
Forest plot of study evaluating the risk factors of iSUA: (**a**) maternal risk factor and (**b**) fetal risk factor.

The results from different analysis methods for evaluating the risk factors of isolated SUA are summarized in Table 2. For iSUA, the overall OR was −0.082 (95% CI, −0.152–0.011, P = 0.023, Fig. 2a) in maternal risk factor analysis, which indicated that pregnant women who had low gravidity were associated with an increased risk for iSUA. No heterogeneity was observed (I^2^ = 0%). As to fetal risk factors, female fetuses were associated with an increased risk for SUA with the overall OR of 0.805 (95% CI, 0.673–0.963, P = 0.017, Fig. 2b), and there was no heterogeneity (I^2^ = 0%). As shown in Table 2, maternal age [OR: 0.039 (95% CI, −0.071–0.149), Supplementary Figure 1A] and smoking status [OR: 1.390 (95% CI, 0.765–2.554), Supplementary Figure 1B] during pregnancy, fetal BMI [OR: −0.165 (95% CI, −0.412–0.083), Supplementary Figure 1C] and maternal parity [OR: −0.069 (95% CI, −0.139–0.001), Supplementary Figure 1D] were not risk factors of iSUA in our meta-analysis.

**Table 2:** Meta-analysis for the risk factor of isolated SUA.

Analysis Model	Analysis Method	Number of studies	Total people	Heterogeneity		SMD				Publication Bias	
				I^2^ (%)	p-value	OR or SMD	95%(CI%)		p-value	Begg	Egger
maternal smoking status	random	4	1607/301776	76.6	0.005	1.390*	0.765	2.554	0.289	1.000	0.993
maternal age	random	14	1610/294852	68.0	0.001	0.039*	−0.071	0.149	0.484	0.276	0.518
maternal gravidity	fixed	6	990/99193	0.0	0.583	−0.082#	−0.152	−0.011	0.023	1.000	0.648
maternal parity	fixed	6	990/99193	0.0	0.750	−0.069#	−0.139	0.001	0.054	0.452	0.380
neonates BMI	random	3	320/35194	64.9	0.058	−0.165#	−0.412	0.083	0.192	1.000	0.605
neonates gander	fixed	3	551/63905	0.0	0.796	0.805*	0.673	0.963	0.017	1.000	0.624

*: Indicates OR. #: Indicates SMD. BMI: Body Mass Index.

### The correlation between iSUA and general neonatal outcomes

**Figure 3:**
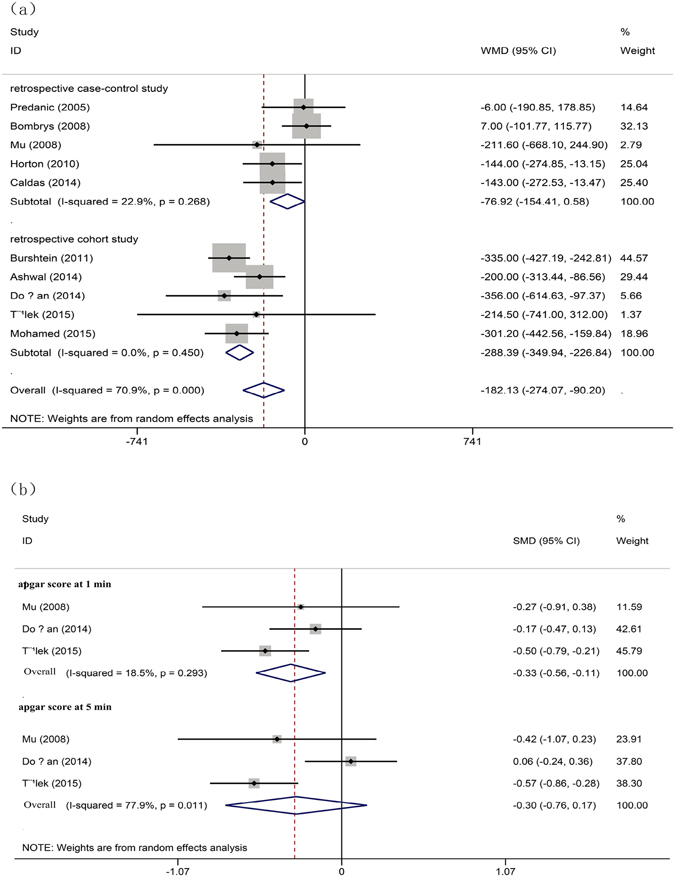
Forest plot of study assessing the association between iSUA and general neonatal outcomes: (**a**) association with birth weight and (**b**) association with apgar score at 1 and 5 min postpartum.

Statistically significant association was detected between iSUA and decreased neonatal birth weight or Apgar score at 1 min postpartum in our meta-analysis. In comparison with neonates with normal umbilical vessels, the iSUA neonates had lower birth weight [WMD for birth weight, −182.132 (95% CI, −274.06–90.198), p = 0.000, Fig. 3a] and lower Apgar score [SMD for Apgar score at 1 min: −0.322 (95% CI, −0.056–0.105), p = 0.008, Fig. 3b] (Table 3). However, there was no difference in Apgar score at 5 min postpartum [SMD: −0.297 (95% CI, −0.76–0.166), p > 0.05, Fig. 3b] between the iSUA group and the normal group.

**Table 3:** Meta-analysis for general neonate outcome between isolated SUA and normal umbilical artery.

Analysis Model	Analysis Method	Number of studies	Total people	Heterogeneity						Publication Bias	
				I^2^ (%)	p-value	SMD or WMD	95%(CI%)		p-value	Begg	Egger
birth weight	random	10	1192/196642	70.9	0.000	−182.132*	−274.06	−90.198	0.000	1.000	0.910
apgar score at 1 min	fixed	3	184/223	18.5	0.293	−0.332	−0.560	−0.105	0.008	1.000	0.868
apgar score at 5 min	random	3	184/223	77.9	0.011	−0.297	−0.760	0.166	0.124	1.000	0.943

*Indicates WMD.

### The correlation between iSUA and adverse neonatal outcomes

**Figure 4:**
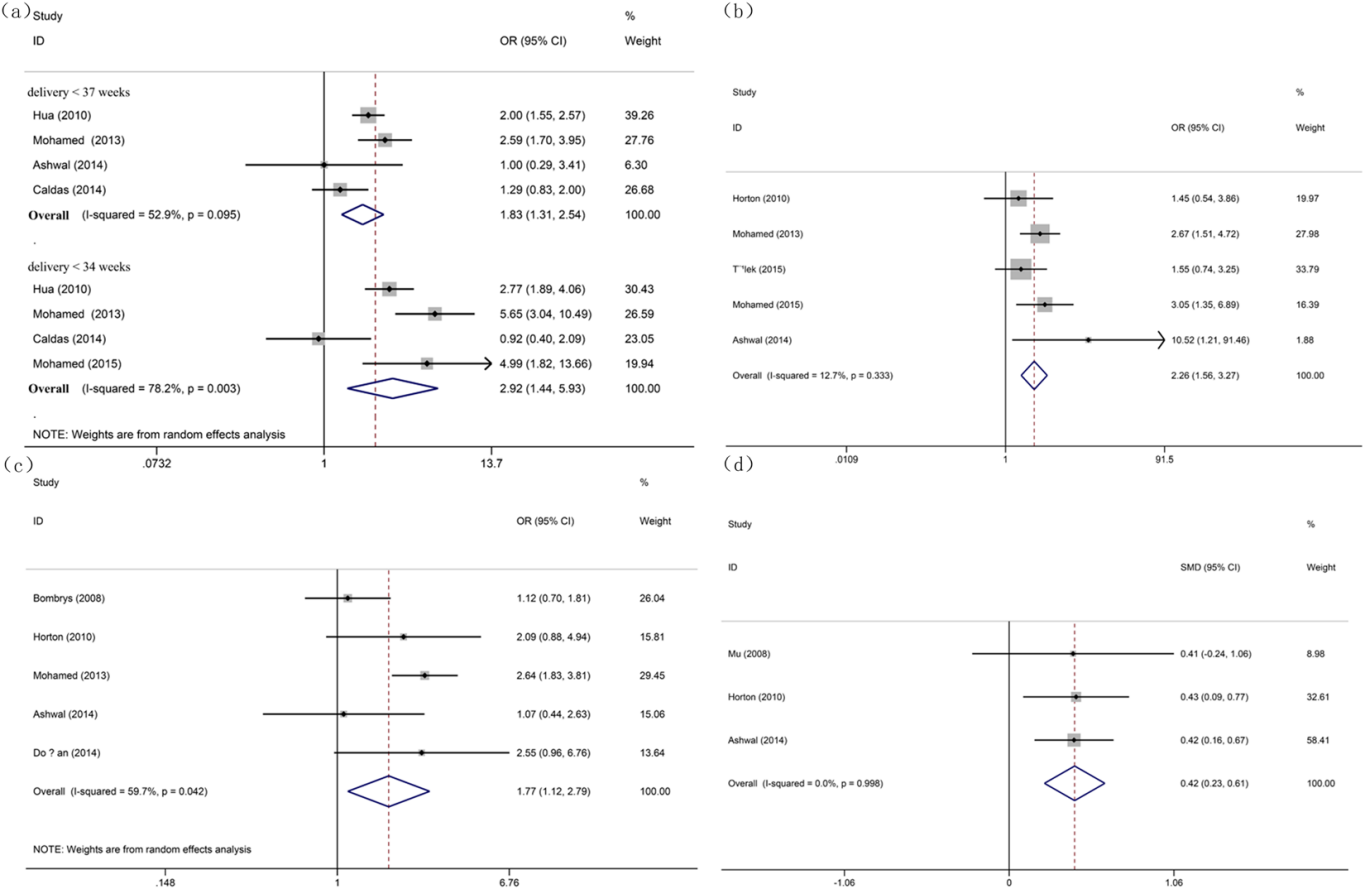
Forest plot of study assessing the association between iSUA and adverse neonatal outcomes: (**a**) neonates of delivery <37 weeks and <34 weeks; (**b**) CS due to fetal distress; (**c**) the rate of admitted to NICU and (**d**) the duration in NICU.

We further analyzed the differential effects of iSUA and normal umbilical artery in adverse neonatal outcomes. Results showed that the iSUA neonates had a significantly higher rate of preterm delivery [OR for neonates of delivery <37 weeks with iSUA: 1.827 (95% CI, 1.314–2.539, P = 0.000); OR for neonates of delivery <34 weeks with iSUA: 2.920 (95% CI, 1.437–5.930, P = 0.003)] (Fig. 4a, Table 4). Thus, iSUA is a risk factor of premature birth.

**Table 4:** Meta-analysis for adverse neonate outcome between isolated SUA and normal umbilical artery.

Analysis Model	Analysis Method	Number of studies	Total people	Heterogeneity		SMD				Publication Bias	
				I^2^ (%)	p-value	OR or SMD	95%(CI%)		p-value	Begg	Egger
Delivery < 37 wk	random	4	776/99622	52.9	0.095	1.827	1.314	2.539	0.000	0.734	0.538
Delivery < 34 wk	random	4	821/99940	71.1	0.008	2.920	1.437	5.930	0.003	1.000	0.968
CS (fetal distress)	fixed	5	547/35876	12.7	0.333	2.258	1.559	3.268	0.000	0.462	0.550
Admitted to NICU	random	5	644/35660	59.7	0.042	1.771	1.123	2.791	0.014	1.000	0.671
NICU day	fixed	3	428/567	0.0	0.998	0.421*	0.227	0.615	0.000	1.000	0.776
Neonatal mortality	fixed	3	961/45139	0.0	0.965	1.749	0.780	3.925	0.175	1.000	0.766

*Indicates SMD. Delivery < 37 wk: neonates born when the gestational age was shorter than 37 weeks. NICU: neonatal intensive care unit. CS: Cesarean section.

Our study also reveals that iSUA was a risk factor for CS because of fetal distress [OR: 2.258 (95% CI, 1.559–3.268, P = 0.000), Fig. 4b] and for the admission to NICU [OR: 1.771(95% CI, 1.123–2.791, P = 0.014), Fig. 4c]. iSUA was associated with prolonged NICU admission [SMD: 0.421(95% CI, 0.227–0.615, P = 0.000), Fig. 4d] (Table 4). On the contrary, iSUA was not a risk factor for neonatal mortality [OR: 1.749 (95% CI, 0.780–3.925, P = 0.175)] (Supplementary Figure 2).

### Assessment of publication bias

The funnel plot, Begg’s test and Egger’s test were utilized to assess the potential publication bias of included studies. The results indicated no evidence of publication bias for all the subgroup analyses (Tables 2, 3 and 4).

## Discussion

SUA is an abnormal condition of the umbilical cord in which one artery is missing. The prevalence of SUA ranges from 0.2% to 11%^1, 9, 15^. Over the past 30 years, numerous maternal and fetal risk factors were reported to be associated with SUA^1, 4–8^. However, due to small sample size and difference in research subjects, previous studies provided limited information and arrived at controversial conclusions. Therefore, a systematic meta-analysis of all available qualified studies may provide us with definitive answers.

Meta-analysis is a powerful approach for investigating the risk factors and neonatal outcomes of iSUA in singleton pregnancy. To date, only 1 meta-analysis publication has focused on the association among iSUA, fetal growth, aneuploidy and perinatal mortality^16^. The results suggested that there was no significant association of iSUA with fetal growth, perinatal mortality or aneuploidy. A large-scale prospective cohort study is needed to reach definitive conclusions on the appropriate work-up in iSUA pregnancies. Thus, we collected a large number of qualified studies for a meta-analysis, aiming to evaluate the risk factors and neonates outcomes of iSUA in singleton pregnancy.

In the current study, we revealed that both maternal status and neonatal sex might be risk factors for iSUA. iSUA itself is a risk factor for Cesarean section and iSUA neonates might have prolonged NICU stay. This result is consistent with the findings of previous reports^1, 6, 14^ that suggested that iSUA was associated not only with anomalies at birth and but also with increased risk of adverse pregnancy outcomes. In our meta-analysis, we found that iSUA neonates had a higher rate of preterm birth < 37 weeks, which suggested that iSUA might be a risk factor for premature birth. Similar results have been published in a previous report^6^. However, our results did not suggest that iSUA is a risk factor for neonatal mortality, which is different from the finding in a previous study in which iSUA was shown to be associated with perinatal mortality. This might be due to a much larger sample size in the current meta-analysis. It should be noted that a meta-analysis^17^ study on a similar issue was recently published after the submission of our manuscript. This study was focused on evaluating the association of iSUA with pregnancy outcomes and perinatal outcomes. No association between iSUA and pregnancy outcomes was identified. iSUA was found to be correlated only with some perinatal complications in this study^17^.

Despite the clinical significance of our study, there are still some limitations. First, selection bias might exist as all studies included in this meta-analysis were published in English. Studies in languages other than English that may have impact on the evaluation were excluded. Second, although all cases and controls in each study were well-defined following the inclusion criteria, there might be factors that were not taken into account but might influence our results if included. Future analyses including more studies on these risk factors are needed to further confirm our findings.

In this article, we presented a meta-analysis to evaluate the neonatal outcomes and possible risk factors associated with iSUA. Our results suggest that maternal primivalidity and the female sex of neonates might be risk factors for iSUA. Fetuses and neonates with SUA and/or iSUA have increased risk of adverse outcomes. Therefore, the diagnosis of iSUA is necessary during pregnancy and attention should be paid to adverse outcomes associated with iSUA for neonates. As such, surveillance with iSUA would improve neonatal outcomes.

## Methods

### Data collection

For the first-round exclusion, articles were searched in the NCBI Global Cross-database, including PUBMED, EMBASE and MEDLINE, using “single umbilical artery” or “two umbilical vessels” or “SUA” and (“fetal” or “prenatal”) and (“Three-vessel cord” or “Normal umbilical cord” or “control” or “two Umbilical arteries” or “3VD”) as key words. Where appropriate, Standard Mean Difference (SMD) or Weighted Mean Difference (WMD) were pooled for the maternal age, gravidity and parity, neonatal birth weight and Apgar score at both one and five minutes postpartum, and odds ratios (ORs) for maternal smoking status, the rate of neonatal delivery before 37 weeks or 34 weeks, Cesarean section (CS) because of fetal distress, the rate of admission to NICU, and the serious adverse neonatal outcome (neonatal mortality to evaluate fetal development as the outcome).

### Inclusion and exclusion criteria

Retrospective cohort studies and retrospective case-control studies were considered eligible if the iSUA described in the study was identified by ultrasound in single pregnancy. In other words, our meta-analysis included all studies on singletons with at least 24 weeks’ gestation and SUA at birth but without identifiable congenital anomalies^4^. SUA was considered to be isolated if there were no additional structural anomalies and markers for an euploidy or small for gestational age (SGA) at the time of the ultrasound scan^15, 16^. Studies were excluded if iSUA was not diagnosed at birth or if the study was only on twin pregnancies. Other exclusion criteria were: presence of any fetal malformation, marker of euploidy by ultrasound examination, chromosomal abnormality established by fetal karyotyping or multi-fetal pregnancy, or absence of a SUA umbilical cord at delivery or by pathological examination^9^.

Possible risk factors included maternal age, gravidity, parity, smoking status, the BMI and neonatal sex. Neonatal data included birth weight, Apgar scores, mode of delivery, rate of preterm delivery (neonatal delivery before 37 weeks or 34 weeks), NICU admissions, and neonatal admission days (length of stay)^11^. Composite adverse outcomes included the following: Cesarean section (CS) because of fetal distress, prolonged neonatal admission and low Apgar score^8^. Neonatal mortality was considered as the serious adverse event.

All studies that are related to the association between the iSUA and neonate’s outcomes or possible risk factors were included. We collected the data of outcomes from all qualified studies for the construction of a 2 × 2 (dichotomous data) or 3 × 2 (continuous date) table.

### Statistical Methods

STATA 12 software was used for all analysis. The overall OR and corresponding 95% CI were calculated for dichotomous data, while the standardized mean difference (SMD) or weighted mean difference (WMD) and 95% CI were calculated for the continuous data. Heterogeneity was measured using the I^2^ index. When I^2^ index was less than 50%, the Mantel-Haenszel (M-H) fixed-effects model was adopted for dichotomous data and Inverse-Variance (I-V) fixed-effects model was used for continuous data. Otherwise, the DerSimonian and Laird (D-L) random-effects model was applied for both dichotomous data and continuous data. A forest plot and Begg’s funnel plot were generated for evaluating publication bias. Higher asymmetry in the funnel plot indicates potential publication biases. To further assess publication bias, Egger’s test was employed for each dataset.

## Electronic supplementary material

Supplementary Info File
