# Supplementary material for: The Risk Factors and Neonatal outcomes of Isolated Single Umbilical Artery in Singleton Pregnancy: A Meta-analysis
Source: Sci Rep. 2017 Aug 7;7:7396. doi: 10.1038/s41598-017-07053-7 (PMC5547151; doi:10.1038/s41598-017-07053-7)

The Risk Factors and Neonatal outcomes of Isolated Single Umbilical Artery in Singleton Pregnancy: a Meta-analysis

Author: Xiaohua Luo[1],Shanshan Zhai[1], Na Shi[1], Mei Li[2], Shihong Cui[1], Yajuan Xu[1*], Limin Ran[1],

Lidan Ren[1], Teng Hong[1], Rui Liu[1]

Affiliations:

1. The Third Affliated of Zhengzhou University, Zhengzhou, 450052, Henan, China.

2. The People's Hospital of Zhengzhou, Zhengzhou, 450000 Henan, China.

***Corresponding author:**

Yajuan Xu

Address: 7th kangfu front street, Erqi District, Zhengzhou, Henan, China. 450052

E-mail:yajuan_hn@sina.com

Business telephone number:+86-0371-66903961

Supplementary figure 1.

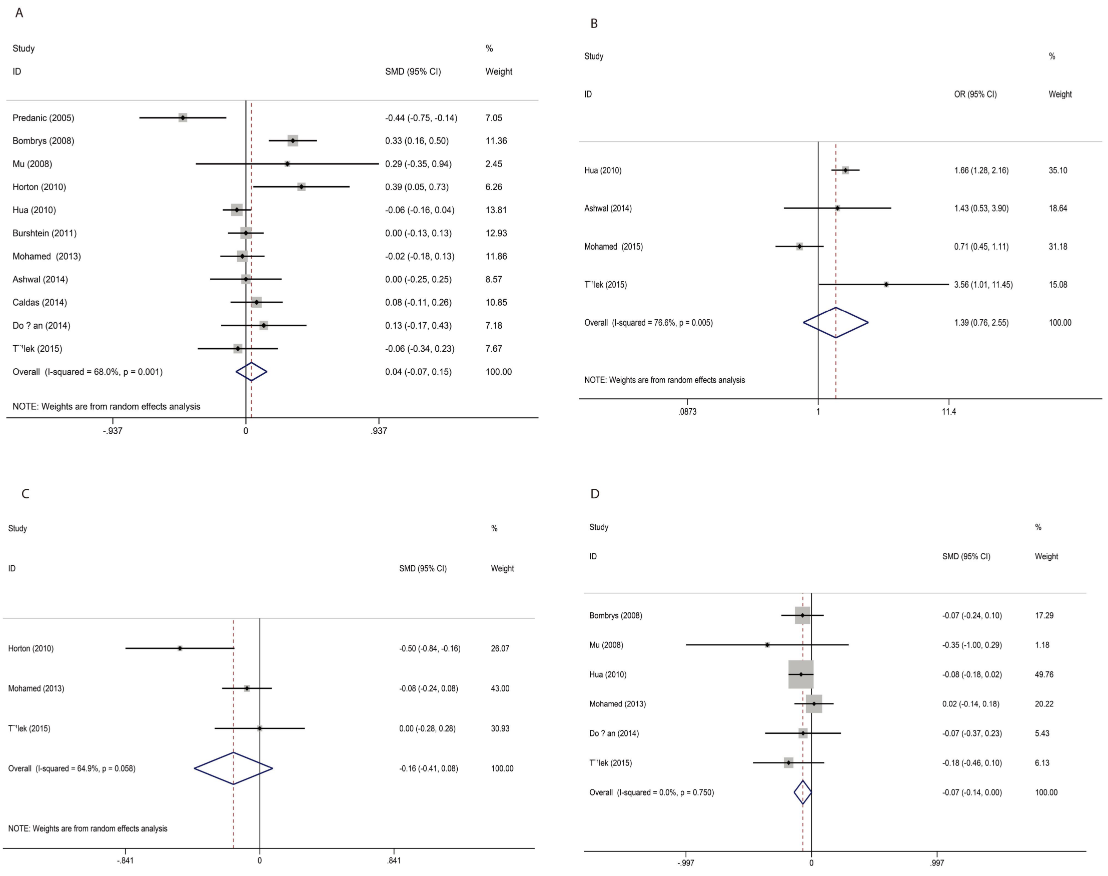

Supplementary Figure 2.

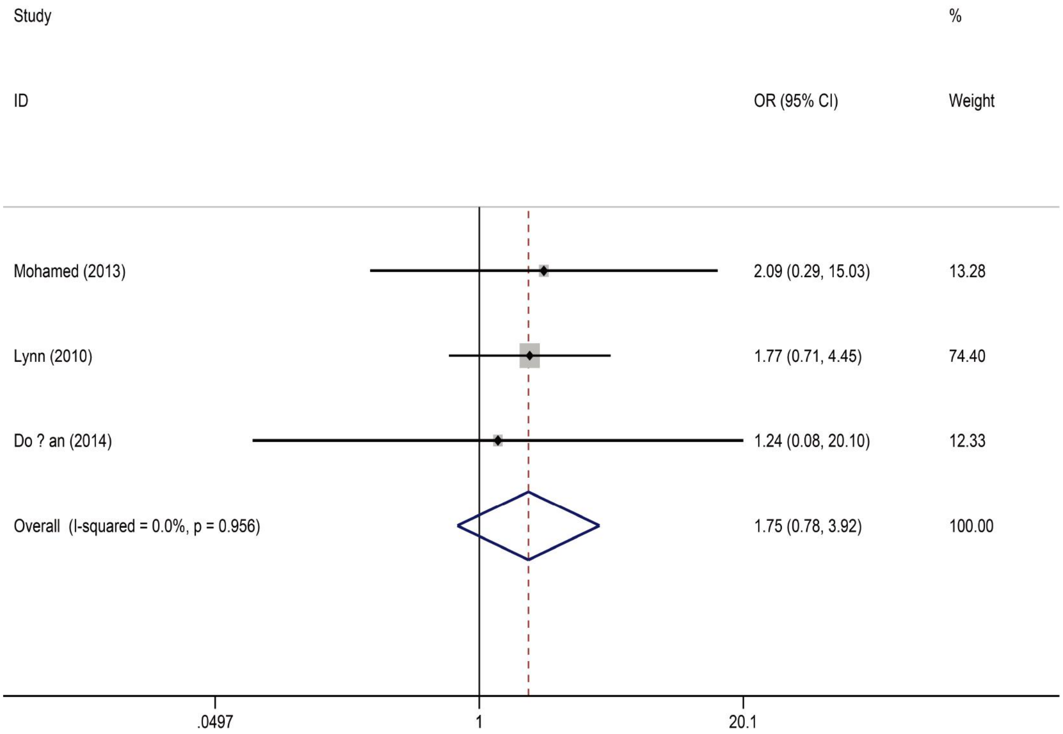

Supplement: Supplementary file 1 — Supplementary Info File [file 41598_2017_7053_MOESM1_ESM.pdf]
